# Supplementary material for: Nanotopographical 3D-Printed Poly(ε-caprolactone) Scaffolds Enhance Proliferation and Osteogenic Differentiation of Urine-Derived Stem Cells for Bone Regeneration
Source: Pharmaceutics. 2022 Jul 8;14(7):1437. doi: 10.3390/pharmaceutics14071437 (PMC9317219; doi:10.3390/pharmaceutics14071437)
Supplement: Supplementary file 1 [file pharmaceutics-14-01437-s001.zip › pharmaceutics-1785521-supplementary.pdf]

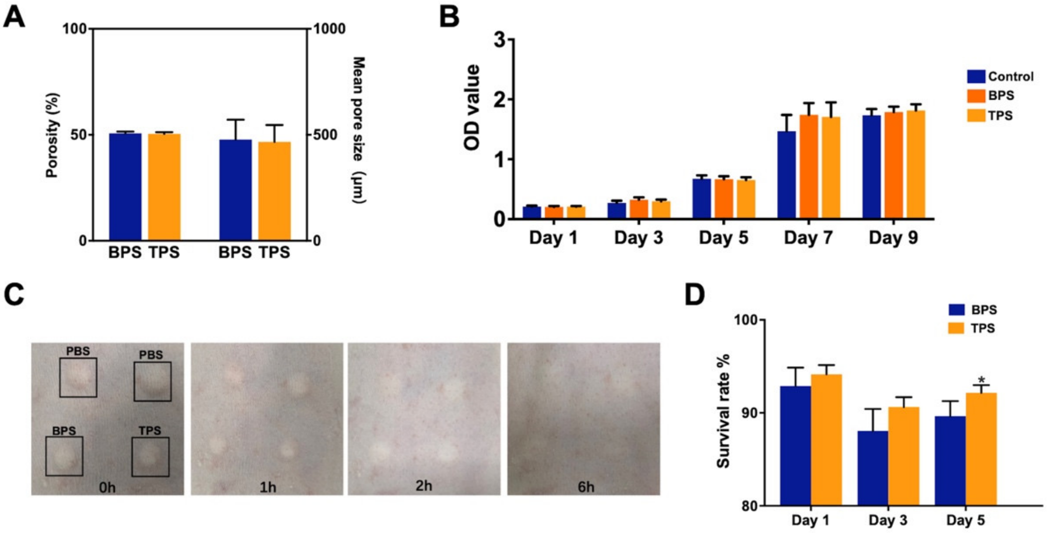

Figure S1 (A) The porosity and mean pore size of BPS and TPS. (**B**) Cytotoxicity test of scaffold infiltration medium. (**C**) Photographs of animals' skin stimulation at 0, 1, 2, and 6 h. **(D)** The survival rates of USCs on the surface of BPS and TPS. * $p < 0.05$.
